# Optimizing Postharvest Edible Coatings for Fruit and Vegetables with Plant-Based Polysaccharides

**DOI:** 10.3390/foods14223897

**Published:** 2025-11-14

**Authors:** Marcos D. Ferreira, Luís E. De S. Vitolano, Fernanda R. Procopio, Ramon Peres Brexó, Larissa G. R. Duarte, Pedro H. B. Nogueira, Vitor P. Bandini, Milene C. Mitsuyuki, Elaine C. Paris

**Affiliations:** 1 Brazilian Agricultural Research Corporation, Embrapa Instrumentation, São Carlos 13561-206, Brazil; nandaprocopio@gmail.com (F.R.P.); ramonperesbrexo@alumni.usp.br (R.P.B.); larissagraziele@gmail.com (L.G.R.D.); milene.corso@embrapa.br (M.C.M.); elaine.paris@embrapa.br (E.C.P.); 2 Institute of Chemistry (IQSC), University of São Paulo (USP), São Carlos 13566-590, Brazil; luis.vitolano@alumni.usp.br (L.E.D.S.V.); pehe_rdp@usp.br (P.H.B.N.); vitor.bandini@alumni.usp.br (V.P.B.)

**Keywords:** polysaccharides, plant-based, fruits and vegetables, starch, cellulose, pectin

## Abstract

Polysaccharide-based edible coatings are increasingly explored as sustainable strategies for maintaining quality of fresh produce, acting as barriers to gas exchange while improving mechanical and optical properties. However, their effectiveness depends not only on the intrinsic features but also on the structural and physiological diversity of fruits and vegetables, which vary in peel composition, hydrophobicity, and texture. This study investigated plant-derived polysaccharide films (cassava starch, potato starch, corn starch, carboxymethylcellulose, hydroxypropylmethylcellulose, and pectin) characterized for moisture resistance, solubility, permeability, thermal stability, hydrophilicity, opacity, gloss, and mechanical strength. Concurrently, different fruits and vegetables (fruit, root, and tubers) were analyzed for their surface hydrophilicity to establish correlations between film properties and peel characteristics. The findings emphasize that no single polymer can be universally applied. In addition, the choice of matrix must be guided by both film functionality and produce surface traits. Starch-based films presented high hydrophilicity, suggesting better wettability, while pectin and cellulose derivatives presented distinct advantages for less hydrophilic peels. This work highlights the importance of tailoring edible coatings according to the physicochemical compatibility between films and fresh produce surfaces, providing insights for improving post-harvest preservation strategies and guiding the development of effective, sustainable coatings for diverse horticultural commodities.

## 1. Introduction

The world faces the challenge of sustainably feeding a growing population, estimated at 9.1 billion people by 2050, reducing food waste and losses in all supply chains, in line with the Sustainable Development Goals (SDGs) and principles of a circular economy. Fruits and vegetables play an important role in the global economy and serve as a dietary staple in most countries [1]. On the other hand, despite all efforts, post-harvest losses and waste remain high, reportedly ranging from 25 to 50% of all produce [2]. Such measurements are difficult to carry out and vary depending on the methodology used. It is generally agreed that, depending on the country and commodity, these numbers are considerably elevated [3]. Additionally, the increasing frequency of disasters caused a loss of approximately 5% of the annual global agriculture Gross Domestic Product (GDP) between 1991 and 2021 [1].

Edible coatings are a low-cost technology that provides multiple benefits, such as moisture and weight preservation, reducing the respiration rate and slowing fruit metabolism, thereby extending shelf-life and decreasing post-harvest losses [4,5,6]. Since edible coatings are composed of major and minor components [7] in direct contact with the produce, food safety must be guaranteed [5]. This is achieved by following regulations set by federal agencies, such as U.S. Food and Drug Administration (FDA) and the European Union which recommend that these materials be Generally Recognized as Safe (GRAS) [8]. Cloete et al. [7] reported an extensive review on edible coatings legislation, showing variations among different countries based mainly in GRAS-approved components. However, some countries, such as Brazil, additionally restrict the use of edible coatings to specific commercialized fresh fruits, i.e., papaya, melon, mango, avocado, pineapple, and citrus, due to skin removal for consumption. On the other hand, Jeevahan et al. [9] reported challenges and issues of transferring edible coatings technology from laboratory to a commercial scale due to distinctive factors. They report the complexity of formulating a coating with sufficient functional features, such as gas exchange and moisture barrier properties, while ensuring all distinctive ingredients and additives are GRAS-approved across various countries, which is critical for produce exportation.

Major components of edible coatings considered GRAS can be divided into two groups: hydrophilic (polysaccharides and proteins) and hydrophobic (lipids) [6,10]. Polysaccharide hydrocolloids are versatile components routinely used in edible coatings. They can be of animal (e.g., chitosan) or vegetable origin (e.g., alginate, cellulose, pectin, starch, carrageenan, etc.) [10] and are mostly recognized as GRAS. However, chitosan, a polysaccharide with a broad range coating application, is not allowed for food contact by the European Union (EU) [8].

The most common polysaccharides of plant origin used in edible films are cellulose derivatives, starch and pectin [11]. Cellulose is the predominant naturally occurring polymer and, due to its strong crystalline structure, is insoluble [12]. Solubility can be achieved by structural modification, obtained from polymers such as methyl cellulose (MC), carboxymethyl cellulose (CMC), and hydroxypropyl methyl cellulose (HPMC), which improve coating properties like moisture barrier and gas permeability [4,13]. Starches are suitable natural polymers for coating formation due to their transparency, good gas barrier properties, ready availability, and low cost [14]. However, their hydrophilic characteristics make them weak barriers against water vapor transmission [4], a limitation that can be minimized by incorporating hydrophobic components, such as lipids. Starch granules are essentially composed of two main components, amylose and amylopectin. Amylose is a straight-chain polymer of α-1,4 anhydroglucose units, with a molecular size ranging from 20 to 800 kg/mol. It accounts for about 20 to 25% of most granular starches and is responsible for the film-forming properties of starch. Structural and molecular weight differences between amylose and amylopectin lead to distinct variations in their molecular and film-forming properties [15,16]. Starches originate from various sources, such as corn, potatoes, cassava, arrowroot, etc. [4], containing different levels of amylose [17]. High amylose content produces films with improved mechanical and barrier properties, due to the more compact structure of linear amylose.

Pectin is the most abundant polysaccharide in the cell wall and middle lamella, especially in citrus peels [18], representing 30% of the cell’s content. Currently, commercial pectin is derived mainly from residues from the processing of citrus and apple peels [15], which have excellent mechanical properties but are highly hydrophilic [4,10]. Kocira et al. [4] point out the potential use of combining pectin and starch to enhance mechanical and gas barrier properties.

The selection of edible coating with suitable features, and composed of GRAS components, that can be applied to a range of fruits and vegetables remains a challenge [9]. Park et al. [19] reported that gas permeation, which regulates gas exchange, is a main property to be considered when selecting coatings.

Edible coatings must establish an appropriate gas composition on a balance between O_2_ and CO_2_. This involves reducing the first one and increasing the second without creating an inadequate atmosphere, with risks causing internal damage and quality loss if that balance is lost [20]. Other parameters, such as wettability, must also be considered in this evaluation [21]. Wettability refers to the capacity of a coating to cover a fruit’s surface. The contact angle is an easy laboratory measurement that quantifies wettability, thereby inferring the hydrophobicity or hydrophilicity of the surface [22]. However, the physicochemical traits of fruit and vegetable skin have mostly been overlooked during edible coating development.

Therefore, the main goal of this study was to characterize plant-based polysaccharide coatings and evaluate their potential application to various fruits and vegetables based on surface characteristics.

## 2. Material and Methods

### 2.1. Material

The polysaccharides used for films production were cassava starch (CA) (Amazon, Manaus, AM, Brazil), potato starch (PS) (MV chemical, Barueri, SP, Brazil), corn starch (CS) (Ingredion, Westchester, IL, USA), citrus pectin (PE) (Perfyl Tech, São Bernardo do Campo, SP, Brazil), carboxymethylcellulose (CMC) (Dinâmica, Indaiatuba, SP, Brazil), and hydroxymethylcellulose (HPMC) (Sigma, San Luis, MO, USA). Glycerin (Alphatec, Lima, Peru) was used as plasticizer. All the other reagents used were of analytical grade.

### 2.2. Synthesis of Films with Polysaccharides and Glycerol

All films were prepared with 2% (*w*/*w*) polysaccharide and with different concentrations of glycerol, 25%, 30%, and 35% (*w*/*w*) [23,24]. At the end of the synthesis, all films were dried in an oven with air circulation at 40 °C for 18 h and then stored in a climate chamber at 25 °C/55% RH for 48 h before characterization. The treatments were named based on the main component and the concentration of glycerol, as described below: cassava starch: CA (25, 30, and 35); potato starch: PS (25, 30, and 35); corn starch: CS (25, 30, and 35); carboxymethylcellulose: CMC (25, 30, and 35); hydroxypropylmethylcellulose: HPMC (25, 30, and 35) and pectin: PE (25, 30, and 35).

#### 2.2.1. Starch Films

##### Cassava Starch Film (CA)

Cassava starch-based films were produced according to the methodology described by Müller et al. [25]. Two grams of cassava starch, 100 mL of distilled water and different concentrations of glycerol were mixed in a beaker. The solution was then homogenized at 80 °C/15 min. Then, the solution was cooled to room temperature (25 °C) and stirred for 20 min.

##### Corn Starch Film (CS)

Corn starch-based films were prepared following the procedure described by Wang et al. [26]. Briefly, 2.0 g of corn starch were dispersed in 100 mL of distilled water in a beaker and heated at 90 °C under magnetic stirring (800 rpm) for 30 min to promote starch gelatinization. After gelatinization, glycerol was added to the mixture, which was then stirred for an additional 10 min to ensure complete homogenization.

##### Potato Starch Film (PS)

The potato starch-based films were prepared according to the method described by Müller et al. [25]. In brief, 2.0 g of potato starch were dispersed in 100 mL of distilled water in a beaker and heated at 80 °C under magnetic stirring (800 rpm) for 30 min to promote starch gelatinization. The resulting dispersion was then cooled to 25 °C, and varying concentrations of glycerol were added. The mixture was subsequently stirred for an additional 1 h to ensure complete homogenization.

### 2.3. Carboxymethylcellulose Film (CMC)

The CMC films were prepared as described by Dong & Wang [27], with adaptations. Film solutions were prepared by dissolving 2 g of CMC in 100 mL of distilled water under magnetic stirring at 70 °C for 45 min for complete dissolution. Then, different concentrations of glycerol were added under magnetic stirring at 70 °C for 10 min.

### 2.4. Hydroxypropylmethylcellulose Film (HPMC)

HPMC films were prepared according to Yao et al. [28]. First, 2 g of HPMC, 100 mL of distilled water, and different concentrations of glycerol were added to a beaker. The solution was kept under magnetic stirring for 30 min at 55 °C.

### 2.5. Citrus Pectin Film (PE)

Pectin film synthesis was processed according to Mendes et al. [29] with modifications. In a beaker under magnetic stirring, 2 g of pectin was slowly mixed with 100 mL of distilled water at 400 rpm for 45 min at 25 °C. Then, glycerol was added in different concentrations and kept stirring for another 15 min at 25 °C. Then, the solution was kept under vacuum to remove air bubbles for 20 min.

### 2.6. Characterization of Films

#### 2.6.1. Thickness and Moisture Content

Film thickness was determined using a handheld digital micrometer (Mitutoyo Co., Kawasaki-Shi, Japan) with an accuracy of 0.001 mm. Measurements were taken at least on five random locations of each film, and the average thickness value was calculated. For moisture content determination, film samples were cut to 2 cm^2^ and weighed before and after drying in an oven at 100 ± 5 °C for 24 h [30]. Water content was calculated using Equation (1).

(1) $$Moisture\;content\;\left(\%\right)=\frac{initial\;mass-final\;mass}{\left(initial\;mass\right)}\times 100$$

#### 2.6.2. Solubility in Water

The water solubility of the films was determined as described by Kavoosi et al. [31], with modifications. To determine the initial dry mass, 2 cm^2^ film samples were cut and dried at 100 ± 5 °C for 24 h. The samples were soaked in 50 mL of distilled water and after 24 h at 23 ± 2 °C, dried again at 100 ± 5 °C for 24 h to obtain the final dry mass. The water solubility of the film was calculated using Equation (2).

(2) $$Solubility\;\left(\%\right)=\frac{initial\;mass-final\;mass}{initial\;mass}\times 100$$

#### 2.6.3. Water Vapor Permeability (WVP)

WVP was determined using an adaptation of the gravimetric method (ASTM E96-92) [32]. Distilled water (1.5 mL) was dispensed into flat-bottomed glasses. Samples of 2.5 cm^2^ were cut from each film and sealed to the bases of the cup, reaching an effective film area of 28.27 cm^2^. The cups were placed inside temperature-controlled BOD incubators at 25 °C with an internal relative humidity of 50%. The cups were weighed every hour for over 8 h using an analytical balance (Model ATX224R, 0.0001 g of precision, Shimadzu^®^, São Paulo, Brazil). Water vapor permeability (WVP) was calculated using Equation (3).

(3) $$WVP=\frac{m\;}{t}\times \frac{x}{A\times ∆p}$$

where WVP is the permeability to water vapor (g·mm/KPa·m^2^·h) calculated from the variation in mass, due to the loss of water vapor (m) that passed through the polymeric material with width (x) and area (A), during the time interval (t) under pressure (Δp) (water vapor pressure difference between the external and internal environment). The analysis was conducted for each formulation in triplicate.

#### 2.6.4. Mechanical Properties

The mechanical properties of the films were analyzed according to the methodology described by Procópio et al. [33]. Uniaxial tensile tests were carried out until film fracture using the Texture Analyzer TA.XT Plus (Stable Micro System, Surrey, UK), with a 50 N load cell and tensile grips at an initial separation of 20 mm. Tests were performed with rectangular samples measuring 50 mm × 10 mm and a crosshead speed of 80 mm/min. Stress–strain curves were plotted for each test and maximum stress (MPa) and elongation at break (%) determined directly from the curves [34]. Six replicates of each treatment were prepared to evaluate each mechanical property.

#### 2.6.5. Differential Calorimetric Scanning (DSC)

Differential Scanning Calorimetry (DSC) was performed using a Q100 Thermal Analyzer (TA Instruments), following the methodology described by Sobral et al. [35]. The analyses employed aluminum crucibles with lids and were performed under a nitrogen atmosphere at a flow rate of 50 mL/min. Samples (5–10 mg) of the raw material and formed films were first heated from 25 °C to 200 °C (10 °C/min), then cooled to 25 °C at the same rate and finally heated again to 200 °C (10 °C/min).

#### 2.6.6. Opacity

The light transmittance of the films was measured at a wavelength of 600 nm using a UV–visible spectrophotometer according to the methodology described by Tan et al. [36], with modifications. Rectangular samples (1 cm × 3 cm) were placed in cuvettes, and an empty cuvette was used as the blank reference. Film opacity was then calculated from the transmittance value at 600 nm using the following Equation (4):

(4) $$\mathrm{Opacity}\;({\mathrm{mm}}^{-1})\;=\;\frac{A_{600}\;}{d}$$

where A_600_ is the absorbance at 600 nm and d is the thickness of the films (mm). Three independent samples per treatment were analyzed.

#### 2.6.7. Gloss Measurements

The gloss meter (Micro-TRI-gloss, BYK Instruments, Columbia, MD, USA) was developed to determine the gloss of edible films, using an incidence reflection of 20°, 60°, and 85°, according to the ASTM D523-89 [37] standard methodology. Gloss measurements were performed five times on the air surface of the films placed in a standard matte black plate, and results were presented as Gloss Units (GU).

#### 2.6.8. Water Contact Angle Test

##### Films

Contact angle analysis was performed following the methodology described by Aloui et al. [38]. The films were evaluated using an optical tensiometer (KSV Cam101, Helsinki, Finland), where sample films (1.5 cm *×* 5.0 cm) were placed on the base. The water droplet absorption was analyzed for 1 min, collecting images every second. The angles between the drop and the samples were calculated by Cam2008 software (KSV Instruments Ltd., Helsinki, Finland). Each formulation was analyzed in triplicate.

##### Fruits and Vegetables

The selection of fresh produce was based on physiological classification and economic importance. Non-climacteric fruits included orange, lemon, and tangerine, representing a group where edible coatings are most frequently applied. Climacteric fruits selected were banana, the most widely commercialized tropical fruit globally, and avocado, a rapidly growing commodity in the international market. Fruit vegetables were divided into non-climacteric (eggplant and green pepper) and climacteric (tomato and cucumber) categories. A root vegetable of significant economic importance, sweet potato, was included, along with the most globally planted tuber, potato. Non-climacteric fruits are more subject to water loss without coating, while coatings on climacteric fruits play an important role in ripening delay. Postharvest losses for root vegetables and tubers can be high, as they are susceptible to both water loss and subsequent rotting. In all cases, the absence of coatings was considered. Samples were cut into a rectangular shape, approximately 2 cm × 5 cm, from three different areas. The tests were conducted using the contact angle test (KSV Cam101). The droplet was analyzed for 60 s, with images collected every second. The angle between the skin and the droplet was calculated using Cam2008 software (KSV Instruments Ltd., Helsinki, Finland). Each fruit or vegetable underwent 5 tests in triplicate, considering the collected skin samples.

### 2.7. Principal Component Analysis (PCA)

Principal Component Analysis (PCA) was performed to reduce the dimensionality of the dataset and to identify correlation patterns among the physicochemical and mechanical properties of the film samples. This multivariate technique transforms the original correlated variables into a new set of orthogonal variables, known as principal components (PCs), which capture the maximum variance within the data [39]. The first principal component (PC1) represents the direction of greatest variance, followed by subsequent components (PC2, PC3, etc.) that describe decreasing amounts of variation while remaining orthogonal to each other. The score plots (e.g., PC1 vs. PC2) were used to visualize groupings and similarities among samples. All data were mean-centered and standardized prior to analysis to ensure comparable weighting of variables. The PCA was conducted using R software version 4.2.3.

### 2.8. Statistics

The effects of polysaccharides materials and glycerol were evaluated using ANOVA and the Kruskal–Wallis test or the Duncan multiple-comparison test, depending on the condition of homogeneity of variance (Bartlett).

The ANOVAs showed significant interactions for most variables, except for gloss (effects of glycerol and polysaccharides material) and thickness (effect of polysaccharides material only). See Supplementary Materials.

The significant interaction led to a large number of treatments in the multiple-comparison tests and, consequently, low power to detect differences. Therefore, it was decided to first compare glycerol concentrations within each polysaccharide material separately, using ANOVA and multiple-comparison tests. The best result was then selected, depending on the desired magnitude of the variable or the group means (maximizing or minimizing effect) when there was no significant difference. All statistical analyses were performed using R software version 4.2.3.

## 3. Results and Discussion

### 3.1. Synthesis Process

The synthesis of films based on potato starch (PS), corn starch (CS), carboxymethylcellulose (CMC), hydroxypropylmethylcellulose (HPMC), and pectin (PE) was achieved using the proposed methodologies. Nevertheless, the preparation of cassava starch (CA)-based films was not successful, as the formulations exhibited high variability and poor structural integrity (Figure S1 in Supplementary Materials), except when low glycerol concentrations (25%) were used. Increasing the plasticizer content led to excessive stickiness, causing the films to shrink and adhere to surfaces, resulting in a glue-like appearance.

Cassava starch possesses highly reactive glycosidic and hydroxyl groups that confer natural adhesive characteristics [33], which were exacerbated by the addition of glycerol. Consequently, film formation was inconsistent and reproducibility could not be ensured. Therefore, due to the inability to obtain films suitable for proper characterization, this formulation was excluded from the final set of analyses.

### 3.2. Thickness and Moisture Content

**Figure 1:**
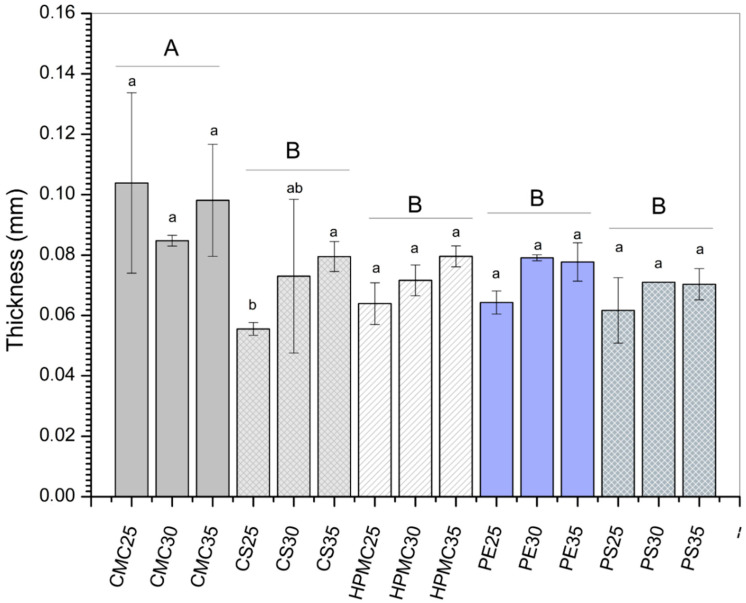
Thickness of films produced from carboxymethylcellulose (CMC), corn starch (CS), hydroxypropylmethylcellulose (HPMC), pectin (PE), and potato starch (PS) with 25%, 30%, and 35% glycerol. Identical uppercase letters indicate no significant difference among polysaccharides formulations, while identical lowercase letters indicate no significant differences across glycerol concentrations within the same polysaccharide group (*p* > 0.05). Comparisons among different polysaccharide formulations were performed using the maximum values when significant differences were observed among glycerol concentrations, or the mean values when no significant differences were detected. The different colors and textures of the bars were used to highlight the results obtained for formulations containing the same polysaccharide, facilitating the visualization of differences among formulations with varying glycerol concentrations.

Considering the effect of the biopolymeric material (expressed as different capital letters in Figure 1), the CMC-based films showed the highest thickness values (0.1 mm), while no statistical difference (*p* > 0.5) was observed for the other polysaccharide matrices (Figure 1). Comparing the increase in plasticizers for each isolated matrix (expressed as different lowercase letters in Figure 1), the effect on thickness was also not observed. Values ranged from 0.06 mm for CS (25% glycerol) formulation to 0.1 mm for CMC (25% glycerol). Other authors have also not identified differences concerning the thickness of polysaccharide-based films [40,41,42]. Variations in film thickness may be related to suspended particles, polymer amount, or free volume [42]. The thickness uniformity found in the present study is essential since this property directly influences mechanical and barrier properties.

**Figure 2:**
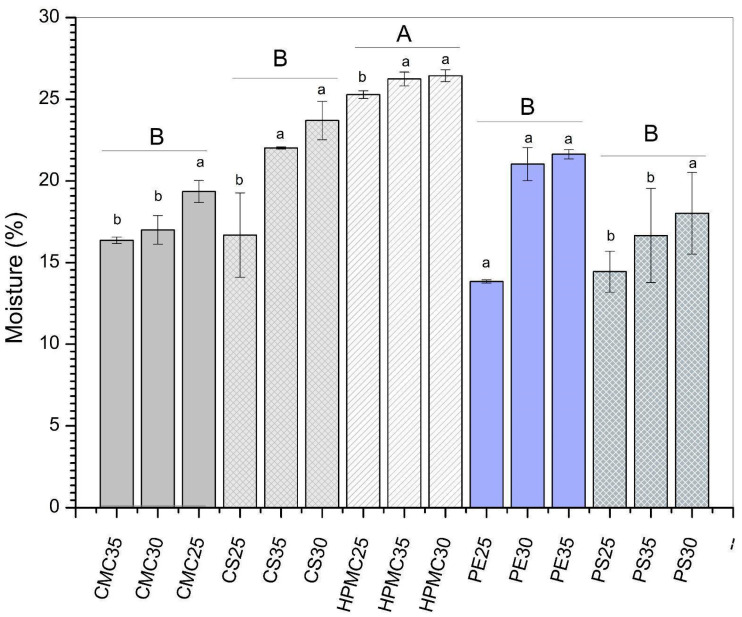
Moisture content of films produced from carboxymethylcellulose (CMC), corn starch (CS), hydroxypropylmethylcellulose (HPMC), pectin (PE), and potato starch (PS) with 25%, 30%, and 35% glycerol. Identical uppercase letters indicate no significant difference among polysaccharides formulations, while identical lowercase letters indicate no significant differences across glycerol concentrations within the same polysaccharide group (*p* > 0.05). Comparisons among different polysaccharide formulations were performed using the maximum values when significant differences were observed among glycerol concentrations, or the mean values when no significant differences were detected. The different colors and textures of the bars were used to highlight the results obtained for formulations containing the same polysaccharide, facilitating the visualization of differences among formulations with varying glycerol concentrations.

The moisture content of the films varied from 13.8 to 25.7% depending on the type of biopolymer used (Figure 2). The highest moisture content was observed for HPMC films (expressed as different capital letters in Figure 2). This behavior can be explained due to the presence of many hydroxyl groups that give HPMC a high moisture absorption capacity [43]. Moreover, the hydrogen bonds between the plasticizer and the polymeric matrix may result in additional space between the polymer chains for water absorption. Regarding the glycerol effect for CS, PS, and HPMC films, the increase in plasticizers caused an increase in moisture content (expressed as different lowercase letters in Figure 2). This effect is common in biopolymer-based films due to the high hygroscopicity of glycerol. Similar results were reported by Luchese et al. [44] for corn starch-based films. In contrast, for the CMC-based films, increasing glycerol decreased the moisture content, while for PE films, increasing plasticizer did not have a significant effect (*p* > 0.05) on the moisture content of the films. This result may indicate good homogeneity of these formulations, since moisture is related to the water absorption capacity of the film-forming matrix during the conditioning period under controlled humidity and temperature conditions [33].

### 3.3. Solubility and Water Vapor Permeability (WVP)

Water solubility is an important parameter for polysaccharide-based films. Lower values are desired to obtain films that are easy to handle and have good resistance to humidity. However, for application as edible coatings into read-to-eat foods, greater solubility will ensure the removal of the protective film before the product is consumed [45].

**Figure 3:**
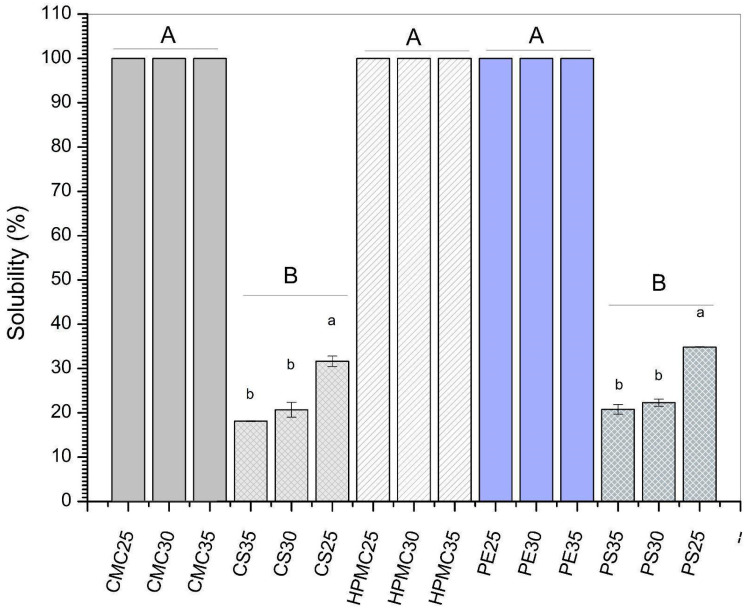
Solubility of films produced from carboxymethylcellulose (CMC), corn starch (CS), hydroxypropylmethylcellulose (HPMC), pectin (PE), and potato starch (PS) with 25%, 30%, and 35% glycerol. Identical uppercase letters indicate no significant difference among polysaccharides formulations, while identical lowercase letters indicate no significant differences across glycerol concentrations within the same polysaccharide group (*p* > 0.05). Comparisons among different polysaccharide formulations were performed using the maximum values when significant differences were observed among glycerol concentrations, or the mean values when no significant differences were detected. The different colors and textures of the bars were used to highlight the results obtained for formulations containing the same polysaccharide, facilitating the visualization of differences among formulations with varying glycerol concentrations.

As presented in Figure 3, except for films based on potato (PS) and corn starch (CS), all other formulations showed 100% solubility in water. Indeed, cellulose and pectin derivatives have greater hydrophilicity than starchy materials. For instance, Mehraj & Sistla [45] observed total solubility for pectin–glycerol blended films. Likewise, Francisco et al. [42] found 93% of water solubility for hydroxyethyl cellulose-based films. Considering the application of these formulations as edible coatings, total solubility may be attractive for fruits and vegetables consumed with the peel, since washing ensures complete removal of the protective film.

Considering the effect of increasing the plasticizer content (expressed as different lowercase letters in Figure 3), potato (PS) and corn starch films (CS) showed lower water solubility (WS) values for formulations containing 35% glycerol. While PS-25% glycerol showed 34% of WS, formulations containing 35% of glycerol presented 20.8% of WS. Similarly, water solubility dropped from 31.6 to 18.1 in corn starch (CS) films with 25 and 35% glycerol, respectively. Generally, the opposite effect is observed since glycerol has a high hydrophilicity. Indeed, Wang et al. [26] observed higher water solubility values when increasing glycerol content in corn starch-based films. Similarly, Ballesteros-Mártinez et al. [46] found that water solubility increased from 23.21% for sweet potato films produced with 10% glycerol to 33.37% in formulations containing 50% plasticizer. Nevertheless, some authors reported that increasing plasticizer content could result in a decrease in free-OH groups [24,47]. This reduction would be related to the formation of stronger hydrogen bonds between starch and glycerol, preventing water from bonding with the film-forming matrix [24]. In this sense, different effects on water solubility can be observed depending on polysaccharide matrices, process conditions, and amount of plasticizers.

**Figure 4:**
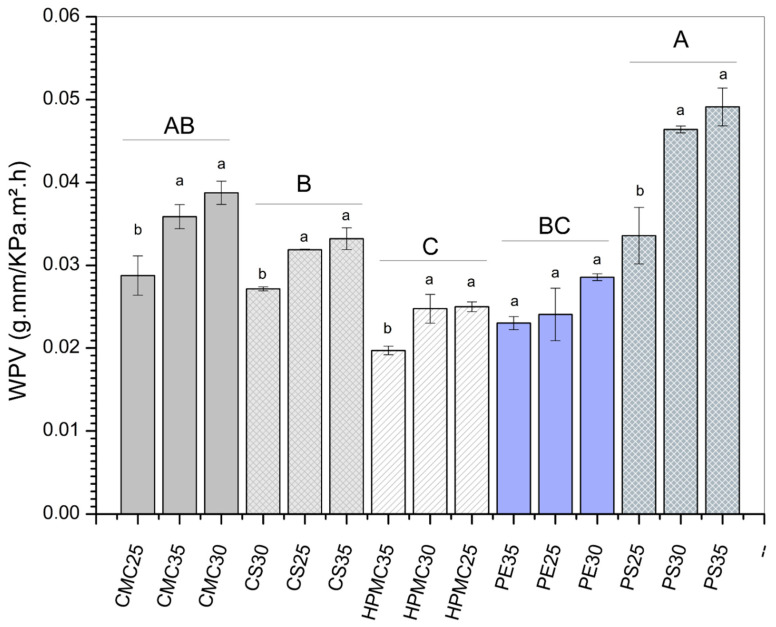
Water vapor permeability of films produced from carboxymethylcellulose (CMC), corn starch (CS), hydroxypropylmethylcellulose (HPMC), pectin (PE), and potato starch (PS) with 25%, 30%, and 35% glycerol. Identical uppercase letters indicate no significant difference among polysaccharide formulations, while identical lowercase letters indicate no significant differences across glycerol concentrations within the same polysaccharide group (*p* > 0.05). Comparisons among different polysaccharide formulations were performed using the maximum values when significant differences were observed among glycerol concentrations, or the mean values when no significant differences were detected. The different colors and textures of the bars were used to highlight the results obtained for formulations containing the same polysaccharide, facilitating the visualization of differences among formulations with varying glycerol concentrations.

The plasticizer content effect (expressed as different lowercase letters in Figure 4) can be better observed regarding the water vapor permeability (WVP) results (Figure 4). For potato starch (PS) and carboxymethylcellulose films (CMC), the WVP clearly increased with increasing glycerol, being 7.09, 12.73, and 11.59 g/ms Pa for PS25, PS30, and PS35, respectively. Similarly, for CMC25, CMC30, and CMC35, values of 4.48, 5.88, and 5.65 g/ms Pa, respectively, were found. Although a barrier to water molecular transport is desirable, some authors [48,49] report that a certain degree of hydrophilicity is desirable when edible coatings are applied to fruits, for example. This behavior is desirable because edible films with moderate WVP can help maintain the fruit’s moisture, preventing accelerated weight loss.

In other formulations, different effects of increasing plasticizer were observed. Corn starch (CS) films showed a first reduction in water vapor permeability when glycerol increased from 25% to 30%. Meanwhile, for the CS35 film (containing 35% plasticizer), the WVP was higher than the other two formulations. In turn, for HPMC-based films, the formulation with the highest concentration of glycerol (HPMC35) demonstrated the lowest water vapor permeability. As for pectin films, samples containing 30% glycerol presented the highest WVP among the three concentrations evaluated.

Analyzing the polysaccharide matrix effect (expressed as different capital letters in Figure 4), HPMC films presented the lowest WVP values, statistically differing (*p* < 0.05) from the others. Several authors reported that WVP is directly related to the quantity of polar (e.g., hydroxyl groups) in the film matrix [40,50,51]. Moreover, the water vapor permeability of flexible films is influenced by the steric distribution of molecules, the presence of pores, empty spaces, and water diffusivity [45]. In this study, potato starch and CMC films showed the highest WVP values. The results agree with other findings for cellulose derivatives [28] and starch-based films [47,51,52,53]. Therefore, it is possible to assume that the ones with high WPV are undesirable for moisture control and may be used for gas exchange and ripening delay [54]. More hydrophilic films generally have this feature. On the other hand, low WPV indicates a most effective moisture barrier. That has a possibility of application in fruits that is desirable to reduce water loss and maintain turgidity. It is a feature of less hydrophilic films).

### 3.4. Mechanical Properties: Stress Resistance and Stretch Percentage

**Figure 5:**
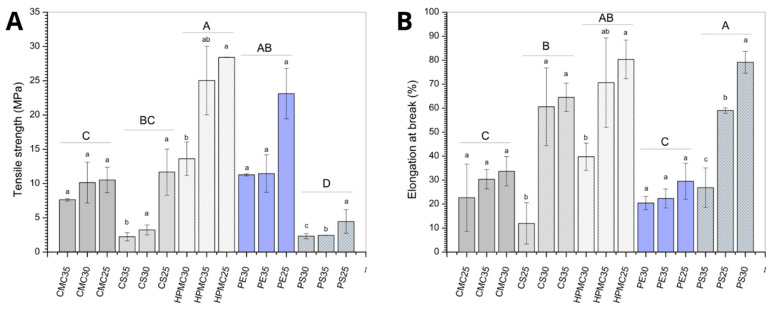
Stress resistance (**A**) and stretch percentage (**B**) of films produced from carboxymethylcellulose (CMC), corn starch (CS), hydroxypropylmethylcellulose (HPMC), pectin (PE), and potato starch (PS) with 25%, 30%, and 35% glycerol. Identical uppercase letters indicate no significant difference among polysaccharides formulations, while identical lowercase letters indicate no significant differences across glycerol concentrations within the same polysaccharide group (*p* > 0.05). Comparisons among different polysaccharide formulations were performed using the maximum values when significant differences were observed among glycerol concentrations, or the mean values when no significant differences were detected. The different colors and textures of the bars were used to highlight the results obtained for formulations containing the same polysaccharide, facilitating the visualization of differences among formulations with varying glycerol concentrations.

Edible coatings can give fruits and vegetables better resistance to mechanical injuries associated with processing, distribution, and handling. In this sense, films with higher tensile strength values are sought to resist minor injuries and higher elongation at break to ensure coating adaptation when damaged without puncturing. Generally, these values are inversely proportional but may change behavior depending on the type of material used. This trend can be observed among the treatments (Figure 5), but the results also indicate an ideal amount of plasticizers to be used to guarantee better tension or deformation properties among the treatments.

Considering the effect of biopolymer material (expressed as different capital letters in Figure 5A,B), potato starch (PS) films presented the lowest tensile strength (approximately 5 MPa) (Figure 5A), while HPMC films showed the higher values (approximately 30 MPa). The greatest elongation at break (Figure 5B) was also observed for HPMC and PS films, with maximum elongation close to 80%. Regarding the plasticizer effect (expressed as different lowercase letters in Figure 5A,B), PS formulations indicate better elastic properties for concentrations of 25 and 30% glycerol, while the 35% formulation reduces this quality, but maintains tensile values. This observation may be an indication that the amount of plasticizer may present an ideal region for addition and that excess glycerol impairs the elasticity of the biopolymer matrix. Similar results can be found Balesteros-Mártinez et al. [46] where higher concentration of glycerol produces higher elongation at break and less rupture forces.

For corn starch (CS) films, the increase in plasticizer content reduced the tensile strength while increasing the elongation at break (expressed as different lowercase letters in Figure 5A,B). The better flexibility due to the higher glycerol concentration may be related to structural changes in the starch matrix. The plasticizer leaves a less dense structure, providing greater mobility between the chains and, consequently, a greater ability to deform before breaking [55]. Nevertheless, polymer–polymer interactions are weakened, resulting in a film that is less rigid and less resistant to tensile stress [56].

The film-forming solutions containing modified celluloses (CMC and HPMC) and pectin (PE) did not show a statistically significant difference (expressed as different lowercase letters in Figure 5A,B) in mechanical properties with increasing glycerol content. The film-forming solutions containing modified celluloses (CMC and HPMC) did not show a statistically significant difference (expressed as different lowercase letters in Figure 5A,B) in mechanical properties with increasing glycerol content. Opposite results were reported in the literature [57,58], where a reduction in tensile strength and an increase in elongation at break were observed as the plasticizer concentration increased. When this behavior is observed, it indicates that the addition of plasticizer fulfilled its role of reducing the attractive forces between the polymer chains. The results observed in the present study, where no effect was found on the mechanical properties of pectin, CMC, and HPMC films by increasing the plasticizer amount, suggest that the polymer–polymer interactions remained stronger.

Overall, HPMC was assumed as the better group result. Potato starch had similar results for elongation at break, and pectin samples presented similar tensile strength values, also being potential alternatives regarding mechanical properties.

### 3.5. Calorimetric Exploratory Differential (DSC)

Overall, the films showed thermal behavior similar to the respective unplasticized control (raw material). For potato starch-based (PS) films, the onset temperature for the endothermic event (T_on_) was reduced from 69 °C to 50.34 °C, 54.61 °C, 55.48 °C for PS25, PS30, and PS35 formulations, respectively (Table 1). Generally, the decrease in melting temperature is associated with weakening polymer–polymer interactions to form polymer–plasticizer interactions and to the increase in polymer chains mobility [59,60].

Nevertheless, different effects can be observed depending on the polysaccharide matrix, type, and concentration of the plasticizer. For instance, Edhirej et al. [61] reported an increase in the thermal transition temperature caused by different plasticizers in cassava starch films. The authors attributed this result to stronger hydrogen bonds between the starch and the plasticizer.

Potato starch (raw material) has its melting point (Ton) at around 70 °C (Table 1). After the film formed, the initial point decreased to around 55 °C. This reduction can be attributed to the bonding of glycerol and water within the sample, as indicated by the higher moisture content in the film. A higher glycerol formulation traps more water molecules, dislocating the initial point of melting [50].

The thermal behavior of corn starch (CS)-based films was affected by increasing glycerol content. CS35 presented the lowest T_on_ (44.74 °C) and the highest ΔT (130.83 °C) between samples (Table 1). In contrast, CS25 showed a T_on_ of 69.7 °C and a ΔT of 110.44 °C. This result was expected as increasing glycerol content increases chain mobility and heterogeneity within the polymer network, thus lowering enthalpy and Ton for amorphous regions, and increasing the transition temperature interval (ΔT) [62]. Similar trends have been reported for starch films plasticized with glycerol, where increasing glycerol concentrations decrease measured crystallinity indices and melting enthalpies while modifying degradation and gelatinization behavior [63].

In contrast to the starch-based films, increasing glycerol content in cellulose-derived films (CMC and HPMC) resulted in a shift in the thermal onset temperature (T_on_) to higher values and a narrowing of the thermal transition range (ΔT) (Table 1). This behavior can be explained by the strong hydrogen-bonding interactions between glycerol and the hydroxyl/carboxyl groups of the cellulose derivatives, which promote a more homogeneous polymer–plasticizer network and reduce microstructural heterogeneity [64]. Similar increases in onset or maximum decomposition temperatures and reductions in transition broadness have been observed for glycerol-containing cellulose films [65].

The lack of a clear trend in the thermal behavior of pectin films with increasing glycerol content (e.g., Ton = 55.8 °C at 25% glycerol; 69.97 °C at 30%; 54.86 °C at 35%) can be attributed to several interacting factors. First, glycerol acts not only as a plasticizer but also as a hygroscopic agent. Therefore, small changes in residual moisture and water–plasticizer partitioning markedly affect thermal transitions and can shift Ton in either direction [57]. Furthermore, glycerol can alter the balance between amorphous and semi-crystalline regions in the pectin matrix. Additionally, processing and drying conditions influence plasticizer distribution and residual solvent content, which further increase sample-to-sample variability [58].

### 3.6. Opacity and Gloss

One of the most important coating features is its ability to attract the consumer’s eyes. Food color is directly assumed as a food-quality attribute, especially when the fruit or vegetable passes through a range of colors while ripening. Any influence on the color aspect could be an issue to consumer choice [66].

**Figure 6:**
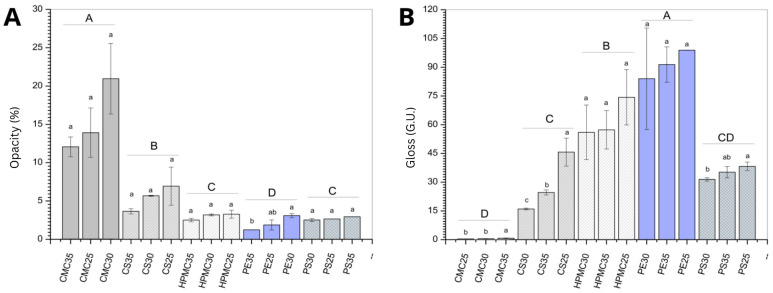
Opacity (**A**) and gloss (**B**) of films produced from carboxymethylcellulose (CMC), corn starch (CS), hydroxypropylmethylcellulose (HPMC), pectin (PE), and potato starch (PS) with 25%, 30%, and 35% glycerol. Identical uppercase letters indicate no significant difference among polysaccharide formulations, while identical lowercase letters indicate no significant differences across glycerol concentrations within the same polysaccharide group (*p* > 0.05). Comparisons among different polysaccharide formulations were performed using the minimum values for opacity and maximum values for gloss when significant differences were observed among glycerol concentrations, or the mean values when no significant differences were detected. The different colors and textures of the bars were used to highlight the results obtained for formulations containing the same polysaccharide, facilitating the visualization of differences among formulations with varying glycerol concentrations.

Considering the biopolymer type effect (expressed as different capital letters in Figure 6A), CMC presented a whitish aspect, reflecting on the highest opacity values. Corn starch film takes the second opaque position, followed by potato starch, HPMC, and pectin. No effect of increased plasticizer (expressed as different lowercase letters in Figure 6A) was observed for any of the biopolymers evaluated.

The higher opacity observed in the CMC-based films, compared to starch-based (corn and potato) and other polysaccharide films (HPMC, pectin), can be attributed to several structural and optical mechanisms. CMC, being a cellulose derivative with abundant hydroxyl and carboxymethyl groups, tends to form highly viscous film-forming dispersions that result in denser and more heterogeneous film matrices [67]. In addition, the presence of residual microcrystallinity in CMC films further inhibits light transmission [68].

Edible coatings not only help preserve food, but can also influence visual characteristics that affect purchase intent. Glossy fruits tend to attract more consumer attention. In this sense, films with a shinier surface can be more appealing when the goal is to attract consumers. Considering the biopolymer effect (expressed as different capital letters in Figure 6B) pectin and hydroxypropylmethylcellulose (HPMC) films exhibited higher gloss values compared with the other tested matrices. High gloss in polysaccharide films is commonly associated with smooth, continuous surface morphology, reduced light scattering, and strong polymer–plasticizer compatibility [69].

The effect of the amount of plasticizer on the surface gloss observed for corn starch (CS) and CMC films may be related to the heterogeneity of the polymer matrix, as previously discussed.

### 3.7. Water Contact Angle Test

**Figure 7:**
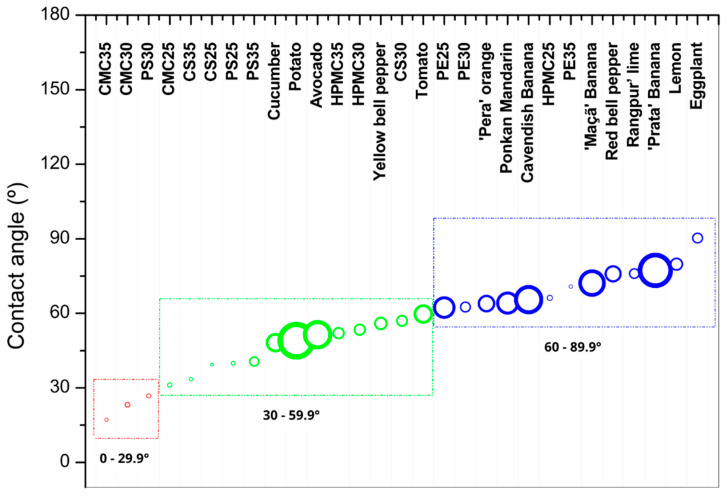
Contact angle measurements of films made from carboxymethylcellulose (CMC), corn starch (CS), hydroxypropylmethylcellulose (HPMC), pectin (PE), and potato starch (PS) with 25%, 30%, and 35% glycerol. The contact angles on the surfaces of various fruits and vegetables were also analyzed, including cucumber, potato, avocado, yellow bell pepper, tomato, ‘Pera’ orange, Ponkan mandarin, Cavendish banana, ‘Maçã’ banana, red bell pepper, Rangpur lime, ‘Prata’ banana, lemon, and eggplant. Circle diameters represent the standard deviation of each sample. Red circles indicate contact angles between 0° and 30°, green circles represent angles between 30° and 60°, and blue circles represent angles between 60° and 90°. Red circle (high hydrophilic, WCA < 30°), green circle (hydrophilic, 30° < WCA < 60°), and blue circle (less hydrophilic).

The water contact angle (WCA) is an attribute associated with the hydrophobicity of the material. Incompatibilities between the hydrophobicity of the coating and the surface of the fruit or vegetable can impair the formation of a protective film. Generally, hydrophilic surfaces have a water contact angle below 90°, while hydrophobic surfaces have values above 90°. Since this is an extensive range, some authors use subcategories to better differentiate wettability properties. For example, films described as superhydrophobic have angles above 150°, while superhydrophilic films have angles below 10° [70,71]. As the water contact angle results ranged between 17 and 70° for films and fruits and vegetables peels, we grouped them into three categories: red circle (high hydrophilic, WCA < 30°), green circle (hydrophilic, 30° < WCA < 60°), and blue circle (less hydrophilic) (Figure 7). All measured water contact angle values for each film or vegetable were presented as Supplementary Materials (Table S1). Sweet potatoes were not included in the table because their rapid water absorption was outside the experiment’s parameters.

CMC-based films were the most hydrophilic biopolymer material (expressed as different capital letters in Figure 7), presenting water contact angles from 0 to 30° (red zone), depending on glycerol content (effect expressed as different lowercase letters in Figure 7). According to Eranda et al. [71], hydrophilic films can exhibit better adhesion to the food surface, in this case, forming a film capable of delaying weight loss. Following the idea of compatibility, films with greater hydrophilicity have greater potential for application to vegetables with similar characteristics, such as cucumber.

Starch films (CS and PS) appear in the second position, showing contact angles from 30 to 60° (green zone). This allows potato and corn starch films to have a wider range of applications, showing a greater propensity to adhere to a larger number of fruits and vegetables.

HPMC-based films were also considered to be hydrophilic, except for the HPMC25 formulation. In this classification, it is less hydrophilic. The lower glycerol content gave the film less hydrophilicity, presenting a contact angle of 60–89°, falling within the blue zone (Figure 7). HPMC25 films that fall within the blue zone appear to have better adhesion to the group of fruits and citrus, allowing for a more integrated relationship with these two categories. According to the study, there are indications that their association will be less relevant to the tested vegetables. However, a higher addition of plasticizer has a beneficial effect on diminishing hydrophilicity, allowing the variation of 30% to 35% glycerol to classify the film as possibly suitable for both fruits and vegetables in the green region (30–59.9°). This makes it the film with the greatest application variability, potentially suitable for all types of fruits and vegetables in terms of adhesion.

The use of films on citrus fruits is frequently applied commercially, using commercial wax dispersions to ensure greater gloss and reduce moisture loss. One of the main reasons for their use of citrus fruits is related to the fact that the peels are not consumed, eliminating the risk of improper consumption of the coating, even if it is food safe. The adhesion of material to the surface can be related to factors such as solvent evaporation, polymerization, absorption, or capillarity, penetrating through pores and filling voids, thus ensuring reduced gas exchange and other benefits sought for fruit coatings and less commonly used for vegetable applications because of the nature of production, being more rotative than fruits. However, on this test, all three citrus fruits were categorized as hydrophilic, and mainly PE35 and HPMC25 (Figure 7) are between the fruit’s surface characteristics leading to the ideal choice for a better and easy association.

On the contrary, CMC and PS30 could only be applied on fruit or vegetables by submersion or with a major material loss because of the difficulty of adherence. While starch-based films, non-washable, and with more hydrophilic characteristics like PS 25, 30, and 35 (39°, 26°, 40°) could be used for coating tubers with similar surfaces, it was observed that sweet potato and potatoes have a high liquid absorption capacity, with the former preventing the measurement of its surface characteristics. There is an indication that any applied liquids will be rapidly absorbed by the tuber, making measurement impossible. Tests must be conducted to determine the most suitable material: a less hydrophilic one, thus creating a more superficial coating, or one as hydrophilic as the peel to fill the pores without encouraging any anaerobic processes.

HPMC leads the greatest range between concentrations of glycerol, taking place on the medium hydrophilic spectrum. Because of that, this material could be used on a bigger range without a selection of different matrices; however, the plasticizer amount will define the application.

The difference in the use of materials can correlate with greater ease of adhesion to the specific characteristics of the fruit, reducing application waste and producing more suitable films with better surface adhesion. However, adjusting other properties such as solubility can ensure their use of vegetables not usually coated due to the impediment to the consumption of their skin.

### 3.8. Principal Component Analysis (PCA)

**Figure 8:**
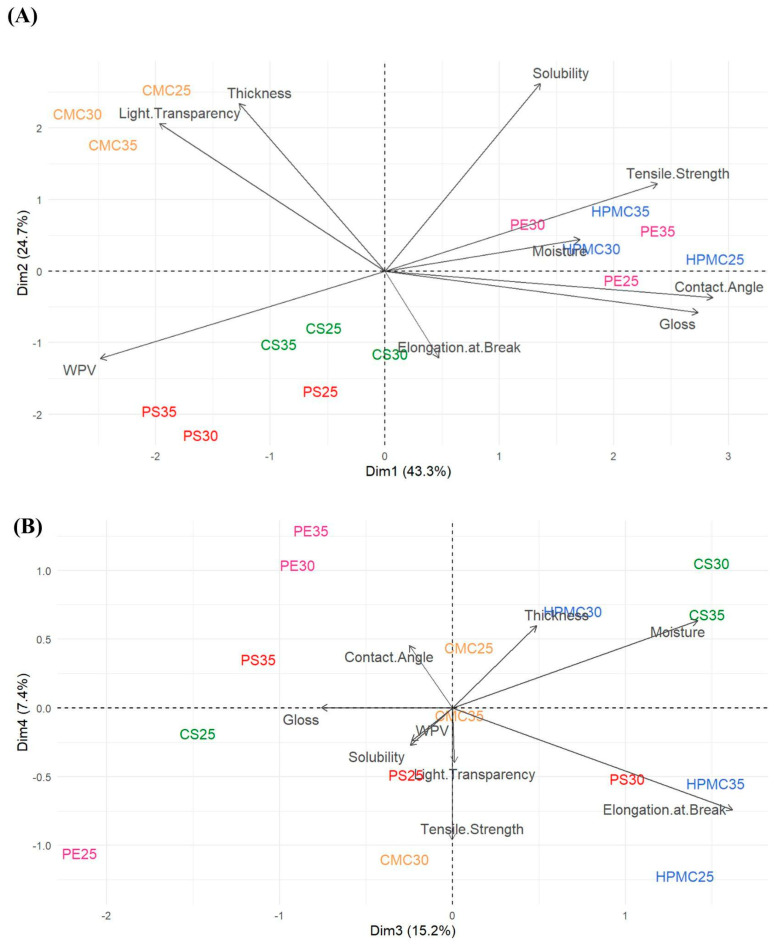
(**A**) Scores of principal components 1 (Dim. 1) and 2 (Dim. 2). (**B**) Scores of principal components 3 (Dim. 3) and 4 (Dim. 4) as obtained through Principal Component Analysis based upon the physicochemical and mechanical properties of the film samples average data of the films: carboxymethylcellulose (CMC), corn starch (CS), hydroxypropylmethylcellulose (HPMC), pectin (PE), and potato starch (PS) with 25%, 30%, and 35% glycerol.

Figure 8 represents the scores using principal components 1 (Dim. 1) and 2 (Dim. 2) (A); components 3 (Dim. 3) and 4 (Dim. 4) (B) for physicochemical and mechanical analyses of average data of the films. The first four dimensions (Dim. 1 to Dim. 4) explained most of the total variance in the data, with the first two (Dim. 1 and Dim. 2, Figure 8A) being the most representative, accounting for a significant portion of the total variance, 43.3% and 24.7%, respectively, for a cumulative total of 68.0%. It is possible to assume that contact angle and gloss are strongly positively correlated, (0.92) and (0.88), respectively. Tensile strength and moisture, in a lower degree, (0.76) and (0.55), respectively. On the other hand, light transparency (opacity) (−0.63) and WPV (−0.79) show strong negative correlation. Therefore, films with larger contact angle, brightness, and mechanical strength, combined with low WPV and optical transparency, decrease hydrophilicity and improve mechanical resistance, such as HPMC25 and PE films. Highest WPV values are associated with low gloss properties and tensile strength, as can be found in starch films, especially in potato films. Therefore, negative quadrant in Dim.1 shows more permeable and transparent samples, then, of a hydrophilic nature. Further, solubility (0.84) and thickness (0.75) are correlated (Dim. 2), showing films with gradient of physical structure, in which thicker and more soluble samples differ from thinner and less soluble ones, representing a physical structure–thickness/solubility axis, as observed in CMC films. The third principal component (Dim. 3) explains 15.2% of the variance, and the fourth principal component (Dim. 4) explains 7.4%, for a cumulative total of 22.6% (Figure 8B). Elongation at break and moisture are correlated, 0.80 and 0.70, respectively (Dim. 3), indicating that this component is more related to the elastic properties and water absorption of materials, reflecting elasticity and water absorption, therefore, more flexible and moist samples, as found in HPMC and corn starch films. Low values in Dim.3 indicate rigid and dry samples. As expected, a negative correlation between film strength and flexibility was shown. Dimension 4 (Dim. 4) showed more dispersed correlations of lower magnitude, suggesting that it explains a residual fraction of the total variance. In the Supplementary Materials Tables S2 and S3 Variance Explained by Each Principal Component, Correlation Matrix Between Original Variables and Principal Components, respectively, and Table S4. Sources of variation by Anova (*p*-values) for physicochemical and mechanical properties.

## 4. Conclusions

Different polysaccharide matrices were evaluated in forming biopolymer-based films to assess the physicochemical and surface properties for applications such as coatings on fruits and vegetables. Furthermore, the water surface interaction of some fruit and vegetable peels was evaluated, looking for possible associations with the elaborate coatings.

The evaluation of this study provided evidence that the analysis of physicochemical properties can be considered to match properties with application objectives. HPMC exhibited the highest water content, as well as high gloss, low water permeability, but was fully soluble. It displayed the best mechanical properties and, based on the contact angle, was considered the most versatile film for application (30–90°), considering only the percentage variation in plasticizer in its formulation. This suggests, based on surface testing with fruits, that its adhesion correlation may be beneficial.

The starch samples showed a relatively low surface angle relationship but covered a broad range (0–60°), allowing their association with a wide variety of vegetables. However, they may not easily correlate with fruits that have less hydrophilic peels. Solubility is a key differentiator of these samples, as they are not fully soluble like the others, allowing for a more durable coating that withstands washing. The potato films demonstrated break resistance comparable to the best-performing group.

Among the polysaccharides evaluated, citrus pectin films fall into the group of the less hydrophilic films (60–90°), depending on their glycerol concentration. However, they exhibit poor mechanical properties and high solubility. Their advantages are related to their ability to prevent water exchange and their high gloss.

There is no evidence that is possible to use one exclusive polysaccharide to accommodate all the needs of coating properties, and other studies must be developed considering the need for water loss, mechanical reinforcement, and mainly skin properties for water interaction to achieve better application, avoiding waste, and ensuring a more complete coating surface. In this study, fruits and vegetables are reported to cover a skin contact angle between 30 and 90°, just as it was investigated for the devolved films. Therefore, the combination of factors related to the natural structure of the fruit must be taken into consideration so that a film is developed to cover the minimum protection needs and to reduce time and difficulties in the horticultural processing chain.

## Figures and Tables

**Table 1:** DSC data of potato starch, corn starch, CMC, HPMC, and pectin. T_on_ (°C) is the initial melting point. T_peak_ (°C) is the maximum melting point and T_end_ (°C) is the final point of melting. ΔH is the enthalpy energy needed to accomplish that transformation. ΔT = T_end_ − T_on_.

Sample	T_on_ (°C)	T_peak_ (°C)	T_end_ (°C)	ΔT (°C)	ΔH (J/g)
**Potato Starch**	69.21	115.8	190.47	121.26	393.7
**PS25**	50.34	105.75	176.54	126.2	146.3
**PS30**	54.61	115.75	177.54	122.93	163.3
**PS35**	55.48	112.37	175.85	120.37	153.8
**Corn Starch**	67.46	115.64	197.26	129.8	307.3
**CS25**	69.7	129.22	180.14	110.44	98.11
**CS30**	58.02	113.81	185.09	127.07	238.9
**CS35**	44.74	91.49	175.57	130.83	55.24
**Carboxymethylcellulose**	92.56	123.31	194.60	102.04	292.7
**CMC25**	56.89	123.42	194.22	137.33	278.4
**CMC30**	58.47	108.32	157.69	99.22	201.1
**CMC35**	71.71	119.39	161.11	89.4	248.1
**Hydroxypropylmethylcellulose**	51.51	100.95	161.49	109.98	111.9
**HPMC25**	33.35	87.01	164.54	131.19	110.7
**HPMC 30**	40.31	90.9	180.90	140.59	73.72
**HPMC 35**	45.51	94.26	148.17	102.66	104.6
**Pectin**	63.69	110.34	179.90	116.21	242.2
**PE25**	55.8	120.7	166.06	110.26	217.5
**PE30**	69.97	123.38	169.48	99.51	241.2
**PE35**	54.86	114.95	176.48	121.62	206.8

(CMC) carboxymethylcellulose; (CS) corn starch; (HPMC) hydroxypropylmethylcellulose; (PE) pectin; and (PS) potato starch. 25, 30, and 35 indicate the glycerol concentration in %.

## Data Availability

The original contributions presented in this study are included in the article. Further inquiries can be directed to the corresponding author.

## Supplementary Materials

The following supporting information can be downloaded at: https://www.mdpi.com/article/10.3390/foods14223897/s1, Figure S1: Visual aspect of cassava starch films containing 25% (A) 30% (B) and 35% (C) glycerol. Figure S2: Thermograms of Potato Starch (A—superior left). Corn Starch (B—superior right). CMC (C—Middle Left). HPMC (D—Middle Right). Pectin (E—Inferior). Base productive in each graphic: Base (Black), 25% of glycerol (Pink), 30% of glycerol (Blue), 35% of glycerol (Green). The line represents the first heat curve and the dot line represents the second heat curve. Table S1: Contact angle values of polymeric films and peels of fruits and vegetables. Table S2: Variance Explained by Each Principal Component. Table S3: Correlation Matrix Between Original Variables and Principal Components. Table S4: Sources of variation by Anova (*p*-values) for physicochemical and mechanical properties.
