# Supplementary material for: Optimizing Postharvest Edible Coatings for Fruit and Vegetables with Plant-Based Polysaccharides
Source: Foods. 2025 Nov 14;14(22):3897. doi: 10.3390/foods14223897 (PMC12651252; doi:10.3390/foods14223897)
Supplement: Supplementary file 1 [file foods-14-03897-s001.zip › foods-3936319-supplementary.pdf]

Supplementary material

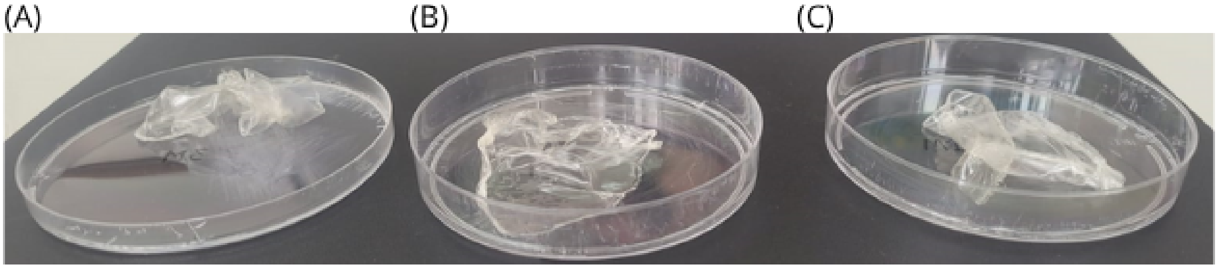

Figure S1. Visual aspect of cassava starch films containing 25% (A) 30% (B) and 35% (C) glycerol.

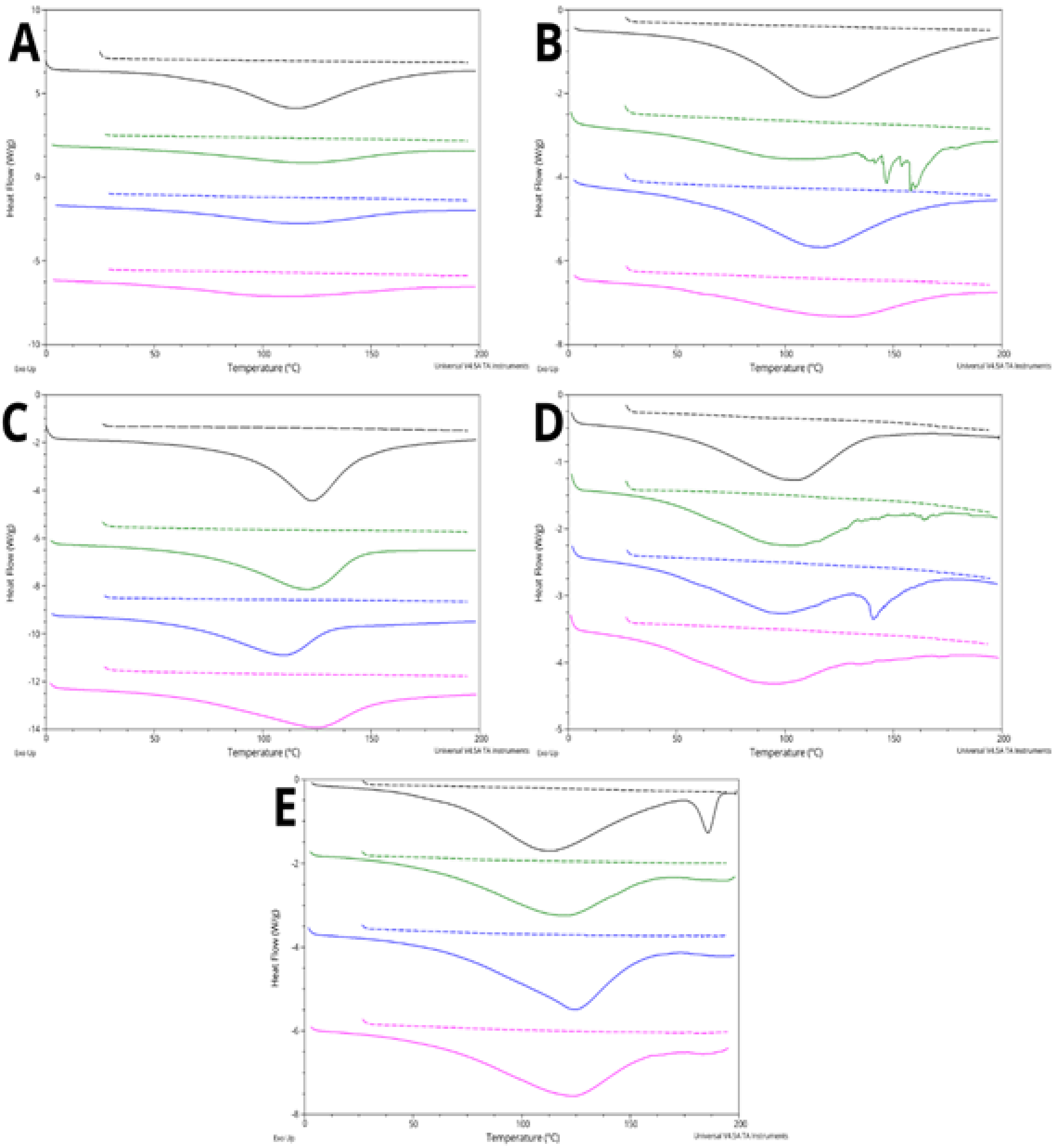

Figure S2. - Thermograms of Potato Starch (A - superior left). Corn Starch (B - superior right). CMC (C - Middle Left). HPMC (D - Middle Right). Pectin (E - Inferior). Base productive in each graphic: Base (Black), 25% of glycerol (Pink), 30% of glycerol (Blue), 35% of glycerol (Green). The line represents the first heat curve and the dot line represents the second heat curve.

Table S1. Contact angle values of polymeric films and peels of fruits and vegetables

Contact angle data							
Films				Peels of fruits and vegetables			
CMC35	17.20	±	4.25	Cucumber	48.20	±	19.77
CMC30	23.20	±	5.80	Potato	49.00	±	38.97
PS30	26.80	±	5.00	Avocado	51.40	±	30.36
CMC25	31.10	±	5.12	Yellow bell pepper	55.90	±	13.69
CS35	33.50	±	4.31	Tomato	59.70	±	19.78
CS25	39.40	±	3.70	“Pera” orange	63.90	±	18.51
PS25	39.90	±	4.67	Ponkan Mandarin	64.10	±	24.05
PS35	40.60	±	10.87	Cavendish Banana	65.50	±	30.66
HPMC35	52.00	±	12.66	“Maçã” Banana	72.20	±	28.55
HPMC30	53.40	±	12.55	Red bell pepper	75.90	±	17.93
CS30	57.00	±	12.32	Rangpur’ lime	76.00	±	11.31
PE25	62.30	±	23.44	Prata' Banana	77.30	±	36.69
PE30	62.50	±	11.65	Lemon	79.80	±	13.75
HPMC25	66.20	±	6.25	Eggplant	90.30	±	11.66
PE35	70.80	±	4.52				

Values are expressed as mean ± standard deviation. Carboxymethylcellulose (CMC). Corn Starch (CS). Hydroxypropylmethylcellulose (HPMC). Pectin (PE). and Potato Starch (PS) with 25%. 30%. or 35% glycerol.

Table S2. Variance Explained by Each Principal Component

	Eigenvalue	Variance Percent	Cumulative Variance Percent
Dim.1	3.9	43.3	43.3
Dim.2	2.2	24.7	68.0
Dim.3	1.4	15.2	83.2
Dim.4	0.7	7.4	90.6
Dim.5	0.4	4.1	94.7
Dim.6	0.2	2.7	97.4
Dim.7	0.1	1.5	98.9
Dim.8	0.1	0.8	99.7
Dim.9	0.0	0.3	100.0

Dim - Dimension

Table S3. Correlation Matrix Between Original Variables and Principal Components

	Dim.1	Dim.2	Dim.3	Dim.4	Dim.5	Dim.6	Dim.7	Dim.8	Dim.9
Contact.Angle	0.92	-0.12	-0.12	0.22	0.06	0.05	0.24	-0.11	-0.04
Gloss	0.88	-0.19	-0.37	0.00	-0.19	0.06	0.02	0.06	0.12
Thickness	-0.41	0.75	0.24	0.30	-0.32	-0.10	0.02	-0.11	0.04
Opacity	-0.63	0.66	0.01	-0.20	0.30	0.09	0.16	0.01	0.07
Solubility	0.43	0.84	-0.12	-0.13	-0.20	0.09	0.05	0.14	-0.07
Elongation.at.Break	0.15	-0.39	0.80	-0.37	-0.16	-0.10	0.13	0.03	0.01
Tensile.Strength	0.76	0.39	0.00	-0.47	0.03	0.05	-0.13	-0.15	0.00
Moisture	0.55	0.14	0.70	0.31	0.13	0.26	-0.09	0.03	0.02
WPV	-0.79	-0.39	-0.12	-0.12	-0.23	0.36	0.03	-0.06	-0.01

Table S4. Sources of variation by Anova (p-values) for physicochemical and mechanical properties

ANOVA (p-values)	Source of Variation		
	Glycerol %	Material	Interaction (Glycerol\ Material)
Contact Angle	0.025	< 0.001	< 0.001
Gloss	0.001	< 0.001	0.312
Thickness	0.152	0.007	0.578
Opacity	0.010	< 0.001	0.024
Solubility	< 0.001	< 0.001	< 0.001
Elongation at Break	0.352	< 0.001	< 0.001
Tensile Strength	< 0.001	< 0.001	0.002
Moisture	< 0.001	< 0.001	0.001
WPV	< 0.001	< 0.001	< 0.001
